# Supplementary material for: Mapping the evolution of fertility support policies in China: A content and instrumental analysis
Source: PLoS One. 2025 Oct 9;20(10):e0332137. doi: 10.1371/journal.pone.0332137 (PMC12510515; doi:10.1371/journal.pone.0332137)
Supplement: S1 Appendix — (ZIP) [file pone.0332137.s001.zip › S1 Appendix. 226 original policy documents/116-人力资源社会保障部、民政部、财政部等关于实施康养职业技能培训计划的通知(FBM-CLI-4-347202).doc]

人力资源社会保障部、民政部、财政部等关于实施康养职业技能培训计划的通知

**发布部门：** [人力资源和社会保障部](https://www.pkulaw.com/law/chl?Aggs.IssueDepartment=60236) [民政部](https://www.pkulaw.com/law/chl?Aggs.IssueDepartment=60208) [财政部](https://www.pkulaw.com/law/chl?Aggs.IssueDepartment=60210) [商务部](https://www.pkulaw.com/law/chl?Aggs.IssueDepartment=60220) [全国妇女联合会](https://www.pkulaw.com/law/chl?Aggs.IssueDepartment=70403) [机构沿革](javascript:void(0))

**发文字号：**人社部发〔2020〕73号

**发布日期：**2020.10.09

**实施日期：**2020.10.09

**时效性：** [现行有效](https://www.pkulaw.com/law/chl?Aggs.TimelinessDic=01)

**效力级别：** [部门工作文件](https://www.pkulaw.com/law/chl?Aggs.EffectivenessDic=XE0303)

**法规类别：** [职业与职工教育](https://www.pkulaw.com/law/chl?Aggs.Category=09123)

人力资源社会保障部、民政部、财政部、商务部、全国妇联关于实施康养职业技能培训计划的通知
（人社部发〔2020〕73号）

各省、自治区、直辖市及新疆生产建设兵团人力资源社会保障厅（局）、民政厅（局）、财政厅（局）、商务厅（局）、妇联：
　　健康照护、养老护理、家政服务、婴幼儿照护等康养服务从业人员职业素质和工作质量，直接关系人民群众日常生活和切身利益。为贯彻落实党中央、国务院有关要求，推进职业技能提升行动，促进康养服务技能人才培养和劳动者就业创业，人力资源社会保障部、民政部、财政部、商务部、全国妇联决定组织实施“康养职业技能培训计划”。现将有关事项通知如下：
　　**一、**总体要求和目标任务
　　（一）总体要求。认真贯彻落实习近平总书记关于统筹做好推进疫情防控和经济社会发展工作的重要指示，围绕做好“六稳”工作，服务落实“六保”任务，聚焦当前人民群众反映强烈的康养服务从业人员数量不足、职业技能水平不高等实际问题，坚持培训先行、人人持证，大规模、高质量开展康养服务人员职业技能培训，健全培养、使用、评价和激励工作体系，加快培养数量充足、素质优良、技能高超、服务优质的康养服务技能人才。
　　（二）目标任务。2020年至2022年，培养培训各类康养服务人员500万人次以上，其中养老护理员200万人次以上；充分利用现有各类职业技能培训和公共实训基地，在全国建成10个以上国家级（康养）高技能人才培训基地；加强职业标准、培训师资和教材建设，不断提升康养培训基础能力。

　　**二、**健全康养服务人员培训体系
　　（三）建立康养服务人员培训制度。建立以地方政府为工作主体，有关部门协调支持，院校、企业、社会团体、职业培训机构、公共实训基地、养老服务机构、儿童福利机构等为实施载体，适应康养服务市场需求和人员就业需要的职业技能培训制度，全面推行康养服务人员就业上岗前培训、岗位技能提升培训、转岗转业培训和创业培训。加强康养服务实训基地建设，支持有条件的企业兴办培训实训机构。扩大职业院校（含技工院校，下同）招生和培养规模，积极面向有意愿从事康养服务的各类人员开展培养培训。
　　（四）全面提升康养服务人员职业技能水平。以提升职业能力为重点，强化康养服务人员的实际操作技能训练、综合职业素质培养，并将法律知识、职业道德、从业规范、质量意识、健康卫生等要求和心理学、营养学等方面的内容贯穿培训全过程。加强失能失智人员照护、老年人照护、康复护理服务、饮食起居照料、生活家务料理、婴幼儿照护、儿童照护、意外伤害预防与处理等方面的岗位技能培训，提升从业人员职业技能水平。
　　（五）健全康养服务培训标准体系。按照健康照护师、养老护理员、家政服务员、育婴员、保育员、孤残儿童护理员等国家职业技能标准、培训大纲和最新行业企业考核评价规范开展培训。针对不同培训对象的文化程度和就业经历，开发全面系统、简便可行的培训课程。对基础性、易操作的知识和技能，组织开发通用速成教材，帮助入职者尽快掌握职业技能要求。有关院校和培训机构要优化课程设置，构建多层次、模块化、高质量的技能课程体系。院校和培训机构要加强校企合作，推行“职业培训包”和“工学一体化”培训模式，增强培训实效。
　　（六）大力培育康养服务企业和培训机构。支持健康照护、养老、家政、托育服务等企业发展，推动建设产教融合型企业。探索以龙头企业和培训机构为主体，支持建设高技能人才培训基地、家政服务职业培训示范基地、家政劳务输出基地等，打造培训实训体系，引领行业发展。鼓励和支持有关职业院校开设健康管理（健康服务与管理）、康复保健、老年保健与管理（老年服务与管理）、护理（养老护理员、育婴员、保育员）、家政服务与管理（家政服务）、公共营养保健等相关专业，强化理论知识和工作实训，加强高层次、综合性专业人才培养。

　　**三、**促进康养服务人员职业发展
　　（七）开展康养服务人员职业技能评价。紧跟新产业、新业态、新模式，加强康养服务新职业和相关职业技能标准开发，做好康养服务技能人才评价工作，畅通从职业资格或职业技能等级五级（初级工）、四级（中级工）、三级（高级工）到二级（技师）、一级（高级技师）的职业发展通道，拓宽职业发展空间。对经评价认定合格的，纳入证书查询系统和信息管理服务平台，向社会公开。推进评价认定信息与相关业务主管部门共享，为康养服务人员培训、使用和监管提供数据支撑。要严格证书管理，加强评价工作的事中事后监管，确保评价质量和效果，防止乱发证和以次充好等不良现象。
　　（八）加强康养服务人员激励保障。加大康养服务人员的激励力度，强化政府激励引导，充分发挥市场机制作用，提升从业人员经济待遇和社会地位，增强职业吸引力。各地、各有关部门开展技能人才评选表彰，可适当向康养服务人员倾斜。鼓励市场主体建立从业人员薪酬待遇与职业技能等级和服务内容、时间、难易等挂钩机制，实现技高者多得，多劳者多得。鼓励家政服务员通过全国家政服务信用信息平台建立信用记录，引导消费者优先选择信用良好的家政服务员。各级政府和有关部门要加强对康养服务人员的职业指导、就业服务和关心关爱，维护其合法权益。
　　（九）广泛组织职业技能竞赛活动。大力开展康养服务人员职业技能竞赛，按照有关规定对获奖选手予以奖励，并晋升相应的职业技能等级。组织开展面向贫困劳动力的职业技能竞赛，将有关康养服务类职业（工种）作为竞赛项目，引导和带动更多贫困劳动力从事康养相关职业。推动世界技能大赛健康和社会照护等项目的竞赛标准转化为职业教育培训标准和课程，促进人才培养与国际接轨，提高培养质量和水平。

　　**四、**做好组织实施工作
　　（十）加强组织领导。各地、各有关部门要充分认识加强康养服务人员职业技能培训的重要意义，把康养职业技能培训计划作为重要民生工程，加强组织领导和统筹实施，建立地方政府主导，人社、民政、财政、商务、妇联等部门协调配合的联动机制，形成工作合力，推进培训信息与相关业务主管部门共享，加强康养服务人员信息信用管理。要将康养职业技能培训计划纳入各地职业技能提升行动，同步进行部署、落实、督导和考核。各地人社部门、民政部门共同做好养老护理员培养培训工作；各地民政部门要做好养老院院长和专兼职老年社会工作者培养培训工作。上述两项具体培训任务由各省（区、市）按2019年底当地60岁以上老年人数量占全国总数比例确定。
　　（十一）加大培训补贴政策落实力度。各地要将健康照护师、养老护理员、家政服务员、育婴员、保育员等作为急需紧缺职业（工种），纳入本地职业技能提升行动“两目录一系统”，按规定落实好职业技能培训补贴、创业培训补贴以及职业技能鉴定补贴政策，所需资金从职业技能提升行动专账资金中列支；按规定落实好生活费补贴政策，所需资金从就业补助资金中列支。有条件的地方，可适当提高职业技能培训补贴标准。各地不得以地域、户籍、年龄、缴纳社保等为前提条件，限制上述人员参加培训并享受培训补贴。
　　（十二）加大宣传和推动力度。各地要采取多种方式，做好培训计划和从业人员待遇政策解读服务。创新方式和手段，开展宣传服务周（月）活动，通过现场咨询、“12333”热线、上门宣传、发放政策宣传页、张贴宣传海报、投放公益广告等方式，提高政策知晓度。要注重培育全国知名劳务输出地，打造康养服务人员劳务品牌，扩大社会影响力。充分利用媒体，选树先进典型，营造劳动光荣的社会风尚和精益求精的敬业风气，不断增强康养服务人员的职业荣誉感和岗位吸引力，鼓励引导更多劳动者技能就业和技能成才。

人力资源社会保障部
民政部
财政部
商务部
全国妇联
2020年10月9日

© 北大法宝：（[www.pkulaw.com](https://www.pkulaw.com/)) 专业提供法律信息、法学知识和法律软件领域各类解决方案。北大法宝为您提供丰富的参考资料，正式引用法规条文时请与标准文本核对。
欢迎查看所有[产品和服务](https://www.pkulaw.com/helps/2.html)。法宝快讯：[如何快速找到您需要的检索结果？](https://www.pkulaw.com/helps/70.html)[法宝](https://www.pkulaw.com/helps/69.html)V6有何新特色？

扫描二维码阅读原文

**原文链接：**<https://www.pkulaw.com/chl/216354eb33f2febebdfb.html>
